# Supplementary material for: Behavior of Regular Insulin in a Parenteral Nutrition Admixture: Validation of an LC/MS-MS Assay and the In Vitro Evaluation of Insulin Glycation
Source: Pharmaceutics. 2022 May 18;14(5):1081. doi: 10.3390/pharmaceutics14051081 (PMC9148014; doi:10.3390/pharmaceutics14051081)
Supplement: Supplementary file 1 [file pharmaceutics-14-01081-s001.zip › pharmaceutics-1665303-supplementary.pdf]

Supplementary data

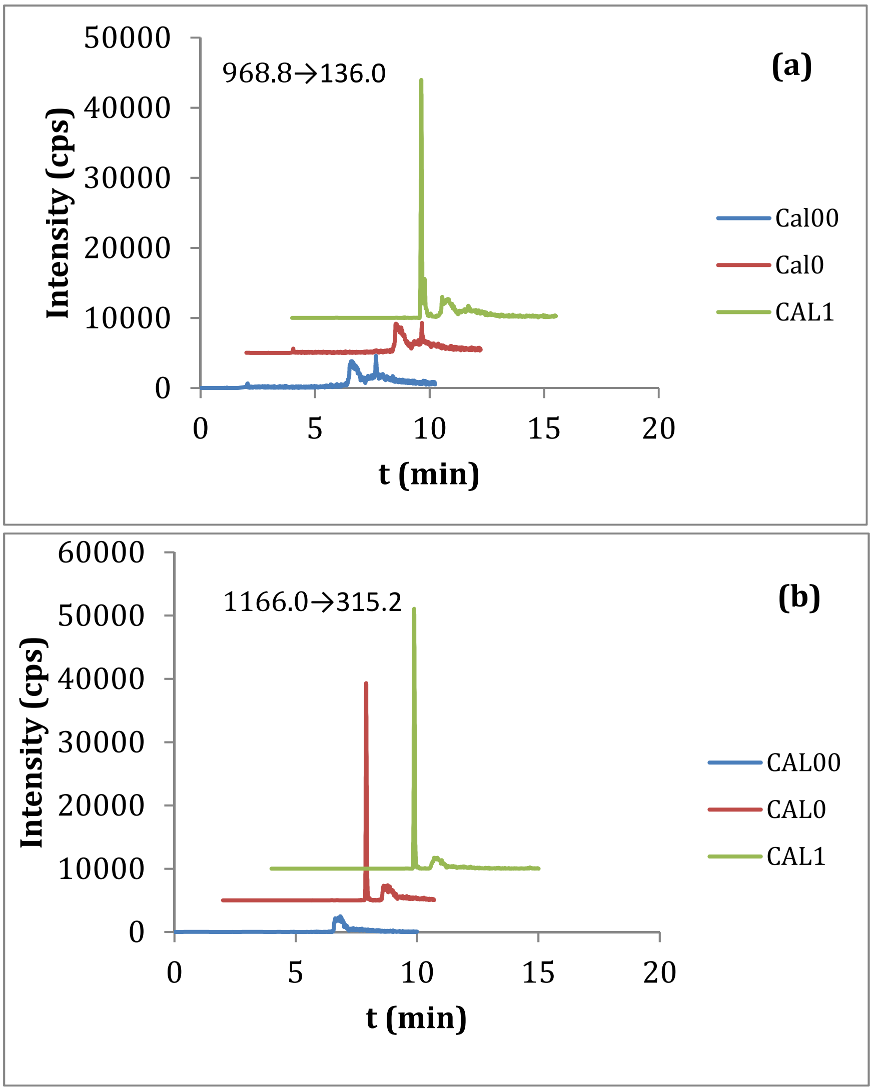

Figure S1: Illustration of the specificity of the method in MRM mode at (a) the R-insulin transition (968.8→136.0) and (b) the IS transition (1166.0→315.2): chromatograms of a matrix blank sample (CAL00), a matrix blank sample spiked with IS (0.060 mg/L; CAL0), and CAL0 additionally spiked with a mixture of R-insulin and glycated R-insulin at a total concentration of 0.016 mg/L (CAL1: $C_{\text{R-insulin}}$ = 0.010 mg/L and $C_{\text{glycated R-insulin}}$ = 0.0060 mg/L) (CAL1).

Table S1: Validation of the LC-MS/MS R-insulin assay: response function, linearity, trueness, and precision.

Validation parameters		
**Response function**[a]		
Concentration range (mg/L)	0.010 – 0.10	
f(X)	Y = 19.72 X + 0.0340	
**Linearity**		
Slope	1.011	
Y intercept	$-7.87.10^{-6}$	
R^2	0.9894	
Trueness	$C_{back-calculated}$ (mg/L)	Relative bias (%)
VS1	0.0099	-0.68
VS2	0.019	-6.01
VS3	0.070	-0.21
VS4	0.100	0.48
Precision	Intraday precision (% RSD)	Inter-day precision (%RSD)
VS1	10.9	10.9
VS2	5.70	6.25
VS3	6.05	8.58
VS4	5.08	5.79

[a] Calibration curves expressing the peak area ratio (R-insulin/IS) *vs*. the R-insulin concentration were obtained from six calibration standards using least-squares-weighted (1/X) linear regression

VS: validation standard; RSD: relative standard deviation
